# Urban thermal comfort trends in Sri Lanka: the increasing overheating problem and its potential mitigation

**DOI:** 10.1007/s00484-022-02328-9

**Published:** 2022-07-19

**Authors:** Shifana Simath, Rohinton Emmanuel

**Affiliations:** The Research Centre for Built Environment Asset Management (BEAM), Glasgow Caledonian University, Glasgow, UK G4 0BA

**Keywords:** Urban heat island, Outdoor comfort, Urban warming, Climate sensitive design, Heat risk

## Abstract

Urban dwellers experience overheating due to both global and urban warming. The rapid urbanisation, especially in hot, humid cities, lead to greater exposure to heat risk, both due to increasing urban populations as well as overheating due to global/urban warming. However, a nation-wide exploration of thermal comfort trends, especially in the hot, humid tropics, remains relatively unexplored. In this paper, we explore the recent historical trends (1991–2020) in outdoor thermal comfort across the entire island of Sri Lanka and evaluate the likely effects of known urban climate mitigation strategies — shade and vegetative cover. We find that ‘very strong heat stress’ is moving towards ‘extreme heat stress’ that was barely registered in 1990s and is now common across two-thirds of the landmass of Sri Lanka in the hottest month (April). Even in the coolest month (January), ‘moderate heat stress’ unknown in the 1990s is now becoming a common trend across the most densely populated parts of the country. High shading and vegetation could reduce heat stress, even in the hottest month, but its utility will diminish as the warming continues in future. As such, policies to reduce global warming needs to be urgently pursued while simultaneously adapting to urban warming in Sri Lanka.

## Introduction

Heatwaves (HW) are consecutive days of extreme temperatures, made worse if associated with high humidity (Im et al., 2017) and air pollution (Lee and Painter, 2015). Their morbidity and mortality consequences are well known (such as the 1995 HW in Chicago, 1998 in Shanghai, 2006 HW in California, and the infamous 2003 European HW that caught the attention of the world, claiming 70,000 deaths — Tan et al., 2007; Robine et al.; Lemonsu et al.; 2015 as cited in Kotharkar and Ghosh 2021). However, much of this information is restricted to developed regions of the world.

At the same time, the increasing global warming is accompanied by strong increases in urban warming in cities (Wang et al., 2021). Given that risk is a function of hazard, exposure, and vulnerability (Cardona et al. 2012), urban heat risk is on the increase due to increasing urban warming (hazard) as well as burgeoning urban population (exposure) (Manoli et al., 2019). In developing cities where poverty and limited adaptive options collide, vulnerability to heat is on the increase as well. This is further exacerbated by the dearth of detailed data, especially in Sub-Saharan Africa, the Middle East, and South Asia (Tuholske et al., 2021).

Although heat stress is a fact of life in the tropics, the consequences of HWs can be deadly even in this region. A study evaluating the impacts of a 2010 Indian heatwave in Ahmedabad, where the temperatures spiked at 46.8 °C, showed that HW contributed to 1334 excess deaths (Azhar et al., 2014). The Ahmedabad heatwave led to heightened awareness and the development of one of the first heat action plans (HAP) in South Asia (Knowlton et al., 2014). The IPCC, in its most recent Assessment Report (AR6), concluded that humid heat stress in South Asia will be more intense and frequent during the twenty-first century (IPCC AR6). The relative share of urban warming, when compared to the total warming, seems to be very high in the South Asian region, reaching up to 76% of the total warming in cities such as Kolkatta, India (Hamdi et al., 2020). There is thus a need to both highlight the scale of the problem as well as explore the likely impact of adaptive actions in the humid tropics.

In this paper, we explore in detail the recent trends (1990–2020) in human thermal comfort in Sri Lanka and highlight the role of some of the well-known urban climate adaptation approaches (in particular, shading, vegetation, and albedo) to moderate the increasingly extreme heat stress. The purpose is to highlight both the urgency of the need and the scale of the adaptive possibility to tackle heat risk in a warm, humid climate where high humidity is already a fact of life, and therefore, mitigation options are limited.

## Background

The importance of urban form to the local climate has long been an area of interest for warm climate researchers (Olgyay, 1963; Knowles, 1981; Givoni, 1998; Martins et al., 2014). The urban form is modulated by the aspect ratio, i.e. the height of the building to the width of the street. Higher aspect ratios result in more shaded streets. However, this can have complications since they tend to trap the radiation and create negative effects (Erell 2008).

Designing buildings in a way to shade the interstitial spaces is a possible approach to enhance outdoor comfort, as recognised by approaches such as the ‘Shadow Umbrella’ concept (Emmanuel 1993). What is needed, however, is an intelligent arrangement of buildings and trees as shading elements to promote both the day- and night-time cooling (Swaid 1992). In terms of other approaches, the orientation of streets and building heights also play an important role. Generally speaking, interstitial spaces along the North–South-oriented streets perform better than the East–West-oriented street, with taller buildings adding considerable coolth at daytime (Jamei et al. 2020). While the latter could interfere with ventilation and daylight, there is evidence that cooling from shading is an overriding factor in the tropics (de Lemos Martins et al., 2016).

Promoting airflow is another approach to thermal comfort enhancement in the tropics. However, the presence of the Inter-Tropical Convergence Zone (ITCZ) in the tropics (where the trade winds meet, and the zone moves with the sun’s annual movement with a time delay) leads to diminished wind movement at the macro-level making it harder to rely on urban ventilation for thermal comfort in the outdoors. Form of buildings (with courtyard form generally preferred (Tablada et al., 2009) orientation of buildings along major ventilation axis (Qaid et al., 2016), a step-up form of building arrangement (Rajagopalan et al., 2014), and a combination of shading and ventilation approaches (Ng and Cheng 2012) could all contribute to enhanced outdoor comfort in tropical cities. Planning approaches such as the well-demonstrated Hong Kong approach called ‘Air Ventilation Assessment’ (AVA) (Ng, 2009) is a useful tool to identify pockets of stagnation and highlight possibilities to facilitate air corridors.

A third approach to overheating management in tropical outdoor is green infrastructure. While parks could provide some localised cooling, their spatial spread is limited in the topics (Chang et al., 2007) unless they are strategically located (Ojeh et al. 2016). The reductions in mean radiant temperature (MRT) achieved by continuous tree cover in streets is a good approach to street-level cooling enhancement (Tsoka et al., 2018) and this is further enhanced by the type of trees (De Abreu-Harbich et al., 2015) and the use of green infrastructure in combination with shading and/or ventilation approaches (Jamei et al. 2020). The cooling effect of green walls and roofs on tropical pedestrian comfort at the street level is modest (Tsoka et al., 2018).

Finally, high albedo materials could contribute to mitigating the urban heat island effect in warm areas and improve thermal comfort in outdoor spaces (Erell et al., 2014), especially in tropical cities where wind speed is low (Emmanuel, 2011). A study by Priyadarsini et al. (2008) indicates that material with a low albedo can increase temperatures up to 2.5 °C. Emmanuel and Fernando (2007) found that high albedo materials can cool the street canyon by 1.2 °C in Colombo, Sri Lanka.

It is clear from the above that a combination of cooling approaches will be needed in the tropics. A further question remains as to the efficacy of these approaches in the face of rapidly deteriorating thermal climate in the tropics. While research on exposure to extreme heat is emerging (Tuholske et al., 2021), the trends in thermal discomfort, a condition that precedes extreme heat exposure but is nonetheless more important to the state of mind and quality of life of urban dwellers, has received less attention in the topics. Additionally, there is a need to study the combined effect of more than one of the urban climate mitigation approaches and their practical applicability to comprehend the maximum positive effects that are possible under a changing climate.

**Fig. 1:**
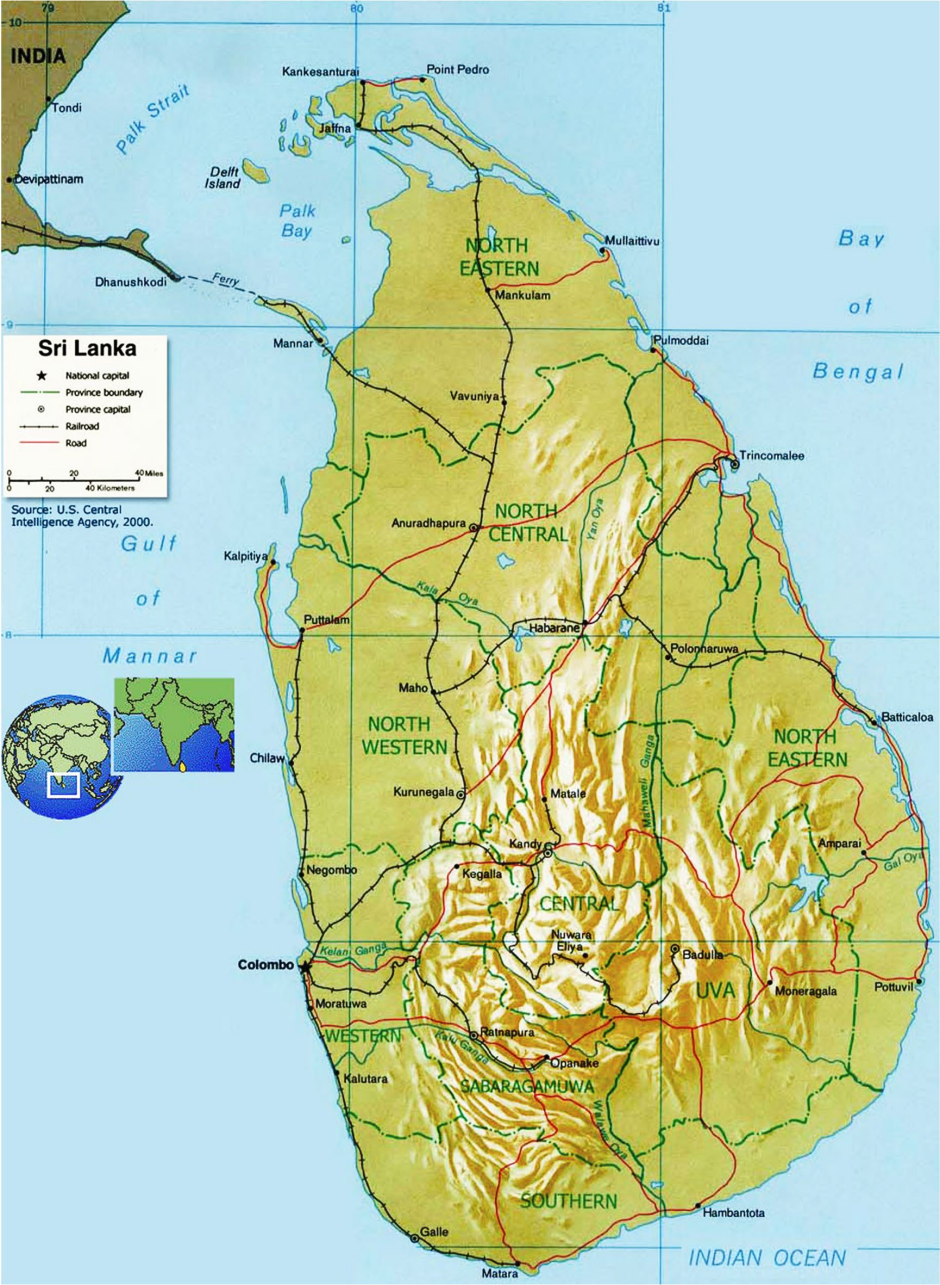
Sri Lanka: location and context. Source: www.asia-atlas.com/sri-lanka.htm

**Fig. 2:**
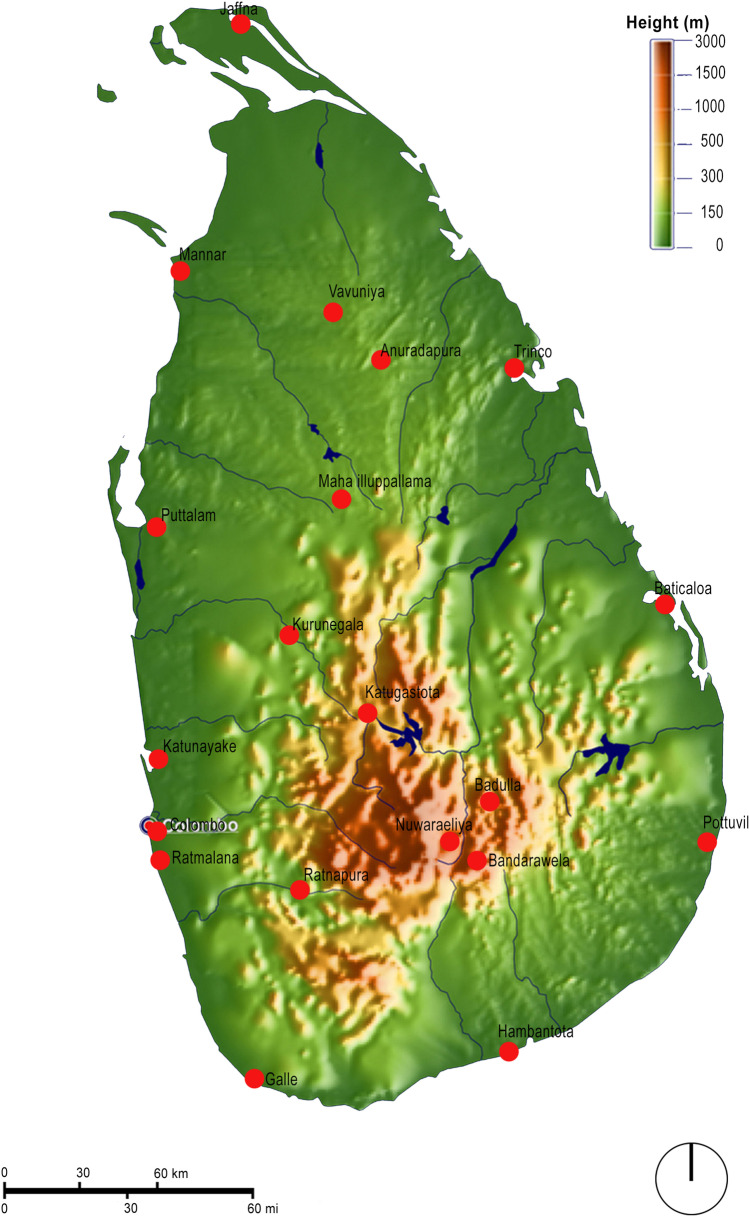
Sri Lanka elevation map. Source: modified from https://www.mapsland.com/asia/sri-lanka/large-relief-map-of-sri-lanka

In this context, an exploration of thermal comfort trends in Sri Lanka is axiomatic in that the overheating issue in a high humidity context could highlight the scale and limitations of the possibilities to manage urban heat. Given its location and climatic context (close to the equator with a hot and humid climate and high solar altitude throughout the year, leading to little seasonal variation except for the monsoon-induced changes in rainfall) as well as the development situation of its capital Colombo (6.9271° N, 79.8612° E) — a primate city representative of many developing nations where a single city dominates the economic context and a coastal city typical of many developing centres in the tropical belt — it offers a useful case study to both highlight the scale of the overheating challenge and the applicability of known approaches to mitigate the challenge (see Fig. 1 for context). In terms of land relief, Sri Lankan coastal areas are nearly flat (less than 150 m MSL) with considerable mountainous areas in the centre (the central uplands, up to 3000 m MSL). Figure 2 shows an elevation map of Sri Lanka which also includes the locations of all of the weather stations used in the present study.

## Methods and materials

In this paper, we first explore the outdoor thermal comfort trends in Sri Lanka over the current meteorological standard reference period for Climate Normal (1991–2020) (World Meteorological Organisation (WMO) 2017). We then explored the likely mitigating effect of known urban climate amelioration techniques.

### Outdoor thermal comfort trends

Climate data from all 20 WMO-approved weather stations in Sri Lanka were obtained from the Department of Meteorology, Sri Lanka. The data consisted of monthly maximum and minimum temperature, maximum and minimum for relative humidity (RH), and the average wind speeds for the current reference period. Initial analysis revealed several missing values, and therefore the period of 1996–2020 was chosen since this period had data from all of the weather stations.

Given the lack of seasonality in this tropical climate, we concentrated our analysis on the ‘hottest’ and the ‘coldest’ months only. We first eliminated outliers using Pauta criterion (i.e. data points larger than 3 standard deviations, Zhu et al. 2004). The maximum temperatures, minimum relative humidity, and average wind speeds were considered for the hottest month analysis and the minimum temperature, maximum RH, and average speeds were considered for the coldest month.

We found that 15 of the 20 stations showed April as the hottest month. Similarly, the coldest month in 17 out of 20 stations was January. As such, the months of April and January were considered for the calculation of human thermal index. This matches with a previous work that indicated the most uncomfortable months in Sri Lanka to be the period between March and May since the solar zenith angle is at its lowest (i.e. the sun is directly overhead), wind speed at its lowest (following the passage of ITCZ), and pre-monsoonal rains contribute to high relative humidity (Emmanuel & Johansson, 2006). Conversely, the solar zenith angle is at its highest, with the northeast monsoon that blows from Northern Asia at its strongest and wind speed highest in January. We used the middle of April and middle of January as the hottest and coldest days for our thermal comfort analysis.

#### Calculation of thermal comfort

Universal Thermal Climate Index (UTCI) is considered the most comprehensive index for calculating heat stress in outdoor environments. It is highly sensitive to weather stimuli (Blazejczyk et al., 2012) and is widely used in outdoor comfort studies (Urban & Kyselý, 2014; Zare et al., 2018). Another widely used outdoor comfort index in the tropics is the Physiologically Equivalent Temperature (PET) (Höppe 1999).

Although we did not calibrate UTCI or other comfort indices to Sri Lankan conditions, several comparable tropical studies exist. Aghamohammadi et al. (2021) proposed a neutral PET value of 24–30 °C for Malaysia. Binarti et al. (2020) reviewed several outdoor thermal comfort indices and evaluated the neutral ranges for hot, humid regions around the world. They too found similar ranges for PET in the tropical belt spanning from South America, West-Central Africa, and South and Southeast Asia. A further review by Potchter et al. 2018) too confirmed these ranges.

We used the ranges given by Zare et al. (2018), which are based on the recommendations of the Thermal Commission of the International Union of Physiological Sciences in 2003. Table 1 shows the UTCI/PET values for corresponding thermal perception. While the neutral temperature in Table 1 is slightly lower than the evidence from the tropics provided in the previous paragraph, the advantage in using the values in Table 1 is their comparability across indices.

**Table 1:** Comparison of thermal perception against different thermal comfort indices. Based on Zare et al. (2018)

Thermal perception	Indices				
	UTCI	WBGT	SET	PMV	PET
Very cold^1^ (extreme cold stress^1,2^)	< 40			− 3	< 4
Very strong cold stress^2^	− 40 to − 27				
Cold^1^ (strong cold stress^1,2^)	− 27 to − 13			− 2.5	4–8
Cool^1,3^ (moderate cold stress^1,2^/moderate hazard^3^)	− 13 to 0		< 17	− 1.5	8–13
Slightly cool^1^ (slight cold stress^1,2^)	0 to + 9			− 0.5	13–18
Comfortable^1,3^ (no thermal stress^1,2^/no danger^3,4^)	+ 9 to + 26	< 18	17–30	0	18–23
Slightly warm^1^ (slight heat stress^1^)				0.5	23–29
Warm^1,3,4^ (moderate heat stress^1,2^/caution^3,4^)	+ 26 to + 32	18–23	30–34	1.5	29–35
Hot^1,3,4^ (strong heat stress^1,2^/extreme caution^3,4^)	+ 32 to + 38	23–28	34–37	2.5	35–41
Very strong heat stress^2^	+ 38 to + 46				
Very hot ^1,3,4^(extreme heat stress^1,2^/danger^3,4^)	> + 46	28–30	> 37	3	> 41
Sweltering ^4^(extreme danger^4^)		> 30			

^1^PET and PMV; ^2^UTCI; ^3^SET; ^4^WBGT.

Accordingly, UTCI values between 9 and 26 °C were categorised as ‘comfortable’/ ‘no thermal stress,’ with 38–46 °C categorised as ‘very strong heat stress’ and > 46 °C is considered ‘extreme heat stress.’ Incidentally, UTCI = 46 °C is equal to Wet Bulb Global Temperature (WBGT) = 30 °C, exposure to which for a day or longer is the ISO occupational heat stress criteria for heat-related illness among acclimatised people performing low to moderate levels of activity (ISO, 2017).

As is well known, thermal comfort is not only a function of environmental variables (air temperature, relative humidity, wind, and mean radiant temperature) but also activity and clothing levels (ISO, 1994). We fixed the non-environmental parameters (clothing level, clo; and activity level, met) at met = 80 and clo = 0.6. The thermal comfort calculations were done in RayMan software (Matzarakis et al., 2010) given its versatility to calculate several comfort indices, including predicted mean vote (PMV), standard effective temperature (SET), physiologically equivalent temperature (PET), Universal Thermal Climate Index (UTCI), and perceived temperature (PT).

In order to derive the spatial trends in outdoor comfort, we then plotted UTCI values in the hottest and coldest months on ARCGIS Arcmap. The maps were created through ArcToolbox > Spatial Analyst Tools > Interpolation > IDW.

### Mitigating the overheating trends

As discussed in the “Background” section, the common urban climate mitigation approaches are as follows: shading, ventilation, green infrastructure, and thermal properties of surfaces. These, in turn, are controlled by the following: aspect ratio, vegetation, wind flow, and albedo.

Given that urban design guidelines applicable in Sri Lanka are uniform across the country and they are based on the guidelines for the Capital, Colombo, we explored the mitigatory effects of known climate control strategies for Colombo only. We used the Urban Development Authority (UDA, 2018) as the basis for creating a ‘typical streetscape’ compatible with the local building guidelines for the base case. We used a 6 m wide street (‘typical street’ under current development regulations) and aimed to maximise the floor space (as denoted by the Floor area ratio = Total building area/plot area) for a 400m^2^ plot.

#### Scenario developments for RayMan simulation

The base case as developed above was then modified by changing the aspect ratio, incorporation of trees, albedo, and street orientations in RayMan (see Table 2 for details). Including the base case, we tested five scenarios for Colombo as a test case to explore the likely cooling effect of urban climate-sensitive design approaches (Table 3).

**Table 2:** Baseline parameters for simulations

*Street*	6 m width and 200 m length, both North–South and East–West orientation streets for all cases
*Tree selection*	6 m height, 4 m crown diameter trees were placed at 4 m intervals (zigzag). Rantzoudi and Georgi (2017) recommends a minimum distance of 2.4 m and more than 5 m high trees are suitable for urban context
*Aspect ratio*	Given the low density, low development status of many Sri Lankan cities, we used a street aspect ratio of 0.5 (i.e. street width is twice that of building height) for the base case and up to 3.0 for other scenarios
*Albedo*	The most common wall and roof materials in use in Sri Lanka are concrete and cladding. Given the excessive rainfall, dark colours (are heavily weatherised surfaces) are the norm. As such, we used albedo = 0.2 for the base case and 0.5 for other scenarios

**Table 3:** Details of different scenarios for RayMan simulations

Base case	Orientation	Aspect ratio	Trees	Albedo	Time of simulation (local time)
Base case	E-W	0.5	No trees	1	13:00 h
Scenario 1	E-W	3	*6 m height, 4 m crown diameter at 4 m intervals placed at zig zag pattern*	0.5	13:00 h
Scenario 2	N-S	3		0.5	13:00 h
Scenario 3	E-W	3		0.5	17:00 h
Scenario 4	N-S	3		0.5	17:00 h

## Results and discussions

**Fig. 3:**
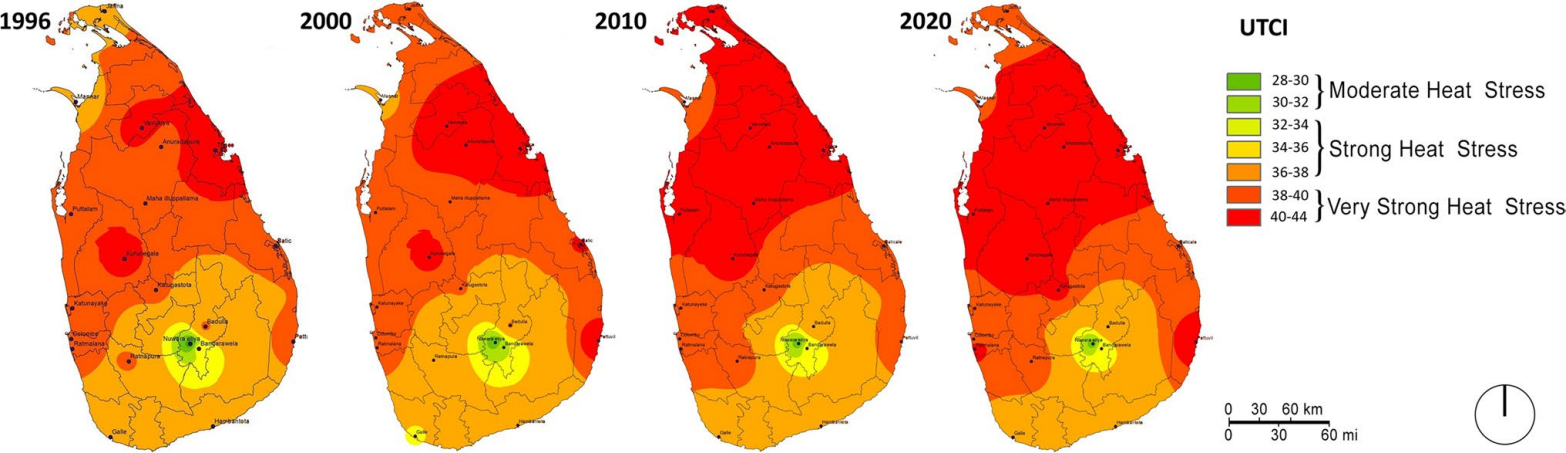
Thermal comfort trends for the hottest month (April)

Figure 3 shows the UTCI trends in the hottest month (April) of Sri Lanka over the study period. Our mapping interpolates the spatial correlation between UTCI ranges.

The Northern region of the country (especially Vavuniya, Trincomalee) showed the highest thermal stress while the central uplands (especially Nuwara-Eliya, Bandarawela) had the highest levels of thermal comfort. Nevertheless, even in the ‘coolest’ region (such as Nuwara Eliya), UTCI had increased from 11.3 to 13.5 °C in 1996–2020. The highest value recorded in Trincomalee for the years 1996 and 2020 were both around 44 $^\circ$ C.

In 1996, 13 out of 20 stations recorded above 38 $^\circ$ C, whereas in 2020, almost all the stations recorded above 38 $^\circ$ C except the central uplands. An increasingly stressful pocket is seen developing in the Southeast of the island (around Pottuvil station) where, for the last 7 years consecutively, UTCI had exceeded 42 $^\circ$ C, which is heading towards extreme heat stress. Similarly, the stations in the Capital region (Ratmalana and Colombo) have reached UTCI = 40 $^\circ$ C. In 2010, nearly half of the stations recorded 40 °C and in 2020 only 6 stations were recorded below 40 $^\circ$ C.

Thus, the entire country is now in moderate heat stress or above, whereas over two-thirds of the country is now in *very strong heat stress* in 2020*.* Furthermore, the capital region as represented by the Colombo/Ratmalana stations (home to approx. 620,000 inhabitants), which was under *moderate heat stress* till 2015, has now moved into *very strong heat stress* in 2020. This has implications for heat risk in that exposure to heat is now more influenced by urban population increase, as recently reported by Tuholske et al. (2021) (in South Asia, the comparative contribution to the increase in the rate of urban exposure to extreme heat is more due to population growth than urban warming).

Considering the fact that UTCI = 46 °C (same as WBGT = 30 °C) is the threshold criteria for occupational heat stress for heat-related illness among acclimatised people, more than two-thirds of the Sri Lankan landmass is now near the threshold of heat tolerance. This amounts to 14.8 million people living in strong/very strong heat stress areas, based on the 2012 census data.

**Fig. 4:**
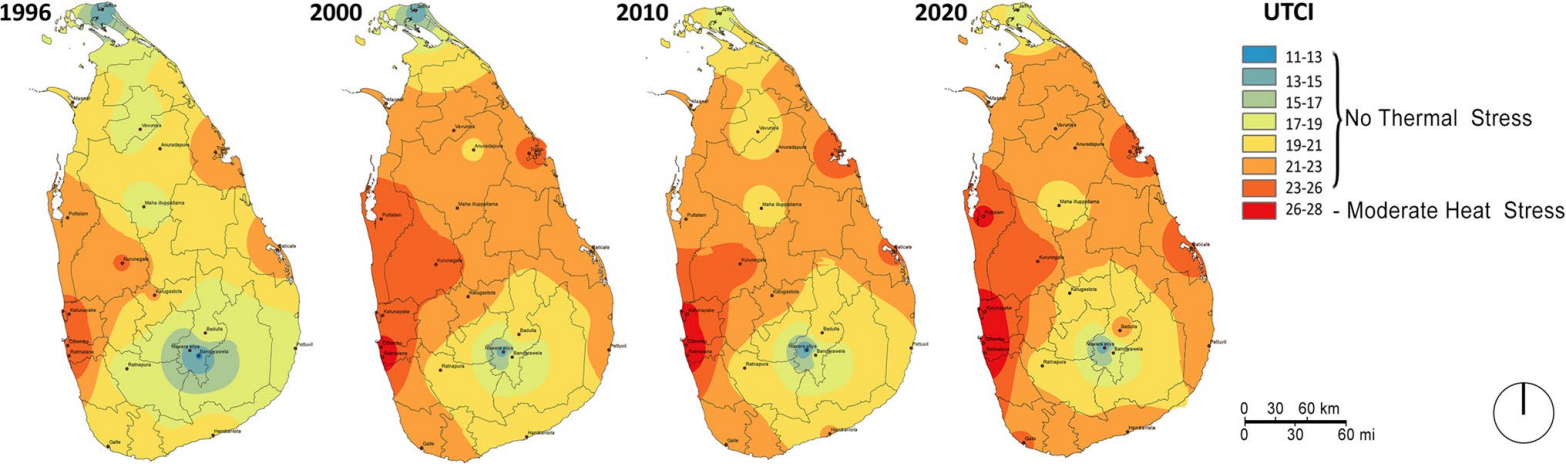
Thermal comfort trends for the coolest month (January)

Figure 4 shows the thermal comfort trends in the coolest month (January). This map suggests that the coolest month is getting warmer throughout the study period, with only the central uplands remaining at 18 °C range. Whereas the entire country was within the *comfort zone or no thermal stress* zone till 2000, parts of the country (especially the Western region around Colombo and Ratmalana) began to reach the threshold of comfort in 2000. Mindful of the fact that January is the peak northeast monsoon season (i.e. wind blowing from the cold North Asian region), the transformation in the Northern and eastern parts of the country in recent years is striking. Even in the central uplands where elevation would normally be expected to provide cool relief, the shrinking of the ‘no thermal stress’ area is remarkable. The other noteworthy aspect is the spread of *moderate heat stress* in the western part of the country, which includes the capital city region.

**Fig. 5:**
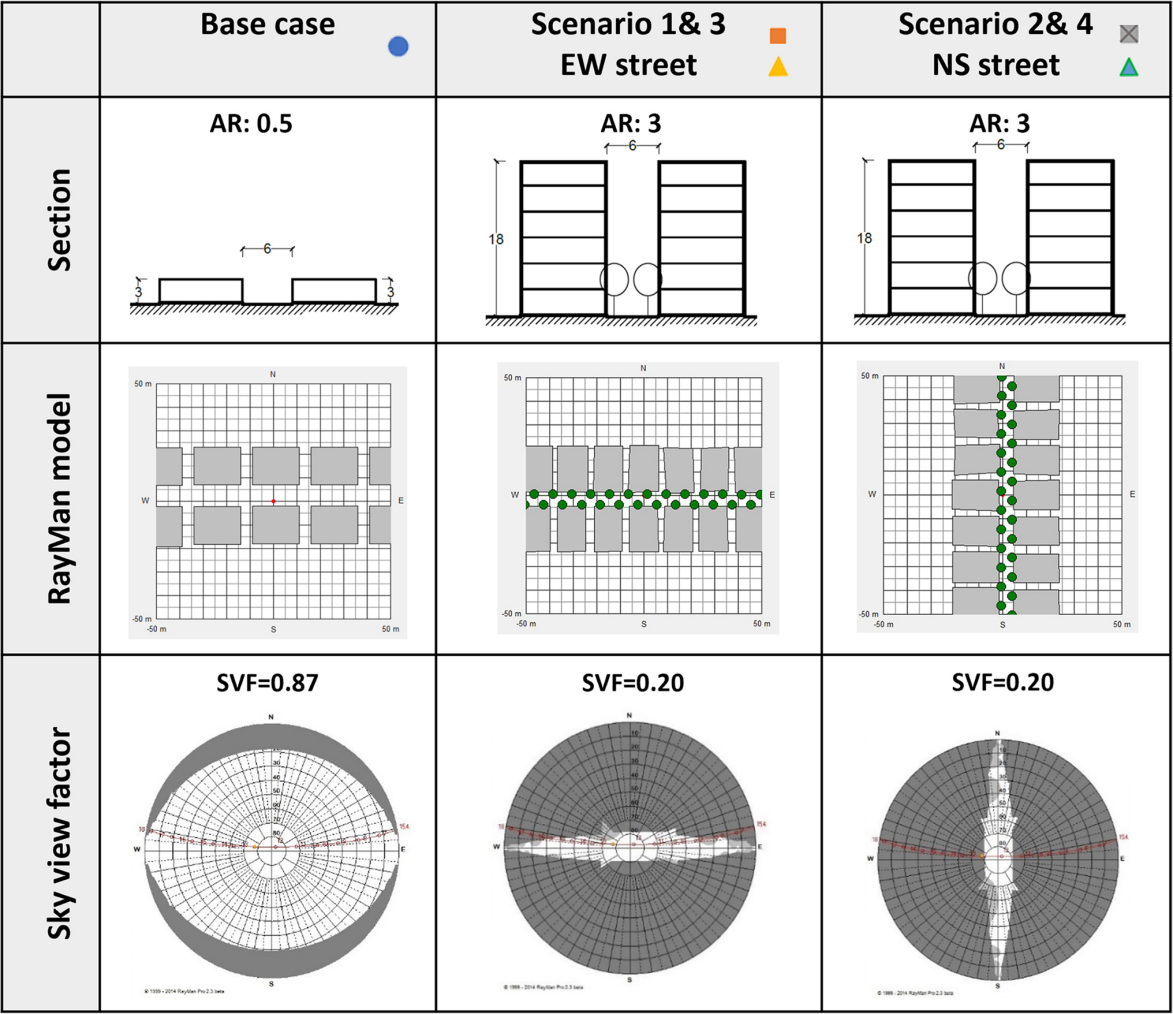
RayMan simulation scenarios

Given this rapid transformation in outdoor comfort in recent years, it is opportune to explore the role, if any, of climate-sensitive design in mitigating heat stress. Figure 5 graphically shows the simulated scenarios.

**Fig. 6:**
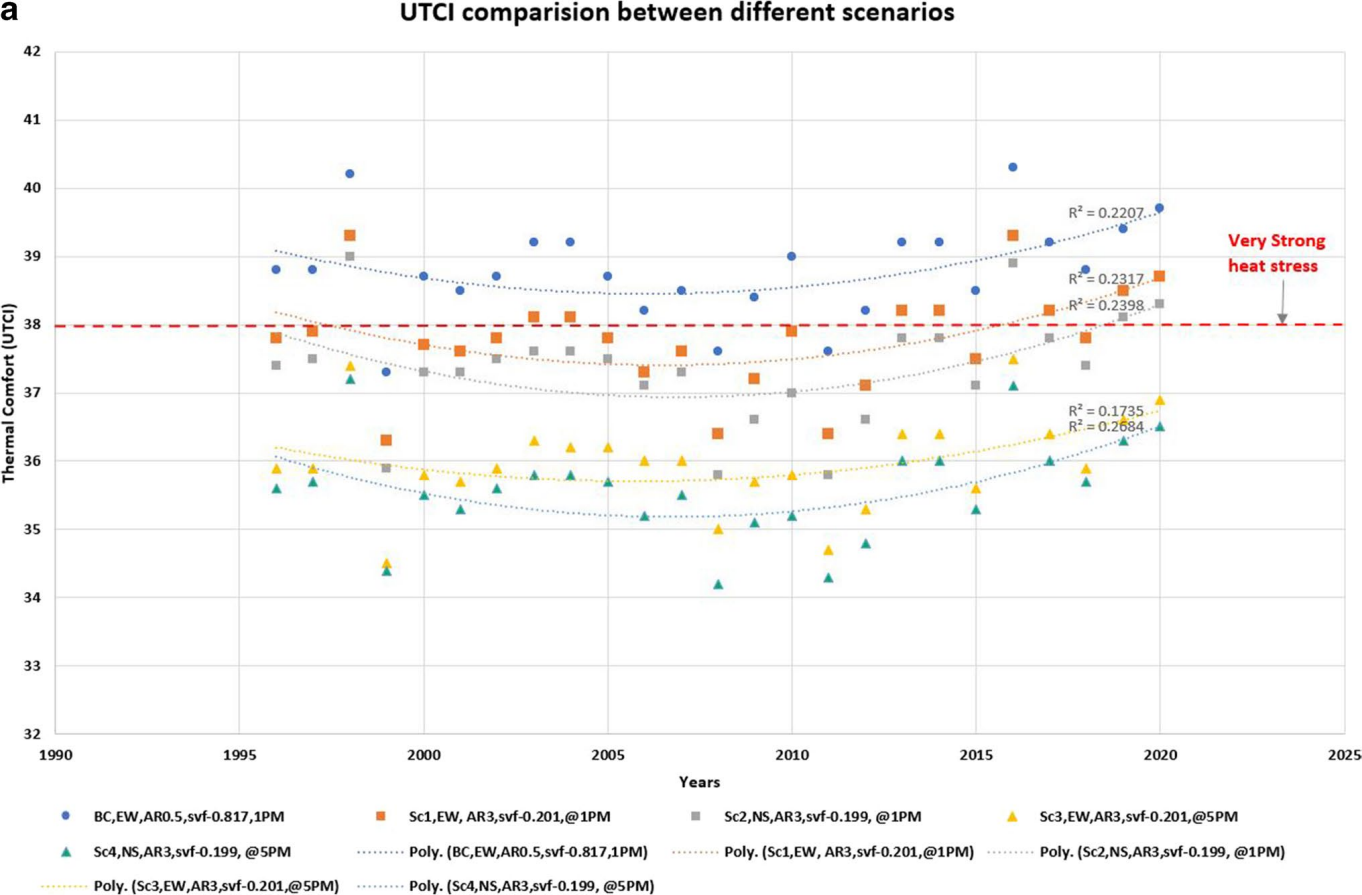

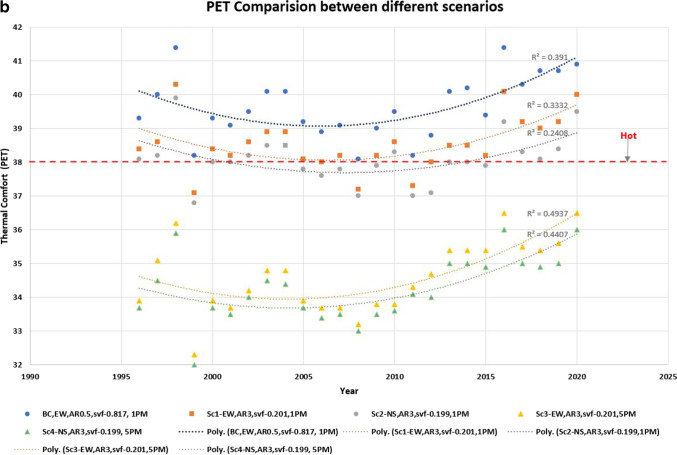
Thermal comfort effects of different scenarios: (**a**) UTCI and (**b**) PET

Figure 6 shows the likely thermal comfort effects of the five scenarios in terms of both UTCI (Fig. 6a) and PET (Fig. 6b). Sc1 and Sc2 (taller buildings in E-W and N-S streets, respectively) show modest improvements to thermal comfort than the base case, with an average reduction of 0.8 °C and 1.1 °C respectively. Sc3 and Sc4 (i.e. taller buildings and vegetation in E-W and N-S streets, respectively) had the best performance, especially in the afternoons, leading to noticeable improvements to thermal comfort (average reduction of 2.7 °C and 3.1 °C respectively). Sc4 showed the best performance out of all scenarios, leading to a consistent reduction in thermal stress (around 3 °C from the base case) and UTCI = 34–36 °C.

The results in terms of PET are even more impressive. An average reduction of 1.1 °C and 1.5 °C for Sc 1 and Sc2 respectively than the base case; results for Sc3 and Sc4 shows an average reduction of 5.03 °C and 5.38 °C, respectively.

In the case of a near-equatorial city such as Colombo, E-W street is continuously exposed to solar radiation while an N-S street is only exposed during noon. By enhancing building-induced shading (i.e. taller buildings) and in combination with street trees, it is possible to reduce thermal stress (from ‘strong heat stress’ to ‘moderate heat stress’), although elimination of heat stress is not possible due to the already high background climate conditions. However, our scenarios 3 and 4 (high H/W ratio) are not compatible with the current building regulations in Sri Lanka, indicating a need to rethink the building regulations from an outdoor thermal comfort point of view.

**Fig. 7:**
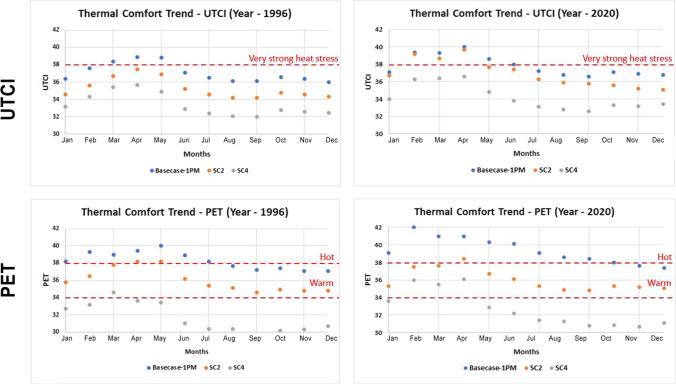
Thermal comfort trends for all months at the start and end of the study period (1996 to 2020)

Figure 7 shows the annual improvements in thermal comfort. In terms of UTCI, Sc 2 would reduce thermal discomfort from ‘Very strong heat stress’ to ‘Strong heat stress’ for two additional months (at 1:00 p.m. local time) while the same scenario would improve the afternoon situation (at 5:00 p.m. local time) for 4 out of 5 months. In terms of PET, Sc 2 would lead from ‘hot’ to ‘warm’ in almost all months except April. This scenario will improve the afternoon comfort even further, where 9 months will move from ‘warm’ to ‘slightly warm.’

However, it is worth noting that the thermal comfort situation in the most recent years (2015–2020) is approaching the limits of tolerance: even the ‘best’ scenario (high shading, N-S orientation with high vegetation) is approaching the threshold of ‘strong heat stress.’

## Implications and conclusions

In this paper, we have shown an increasing trend in thermal stress in Sri Lanka. The trend has increased in severity in recent years. While the Northern and Eastern regions of the country have seen the sharpest increase, the most worrying in terms of heat risk is the Western region which is home to Sri Lanka’s sole metropolitan region (Colombo) with a rapidly increasing population. In 1981, the Western region had a population of 3.8 million, which increased by more than 60% in 2020 to 6.2 million (Department of Census and Statistics Sri Lanka, 2021). The share of the vulnerable population is 38.7% (15% elderly and 23.7% children), which would mean an even higher heat risk, as a function of hazard — increasing heat hazard due to both global and urban warming; exposure — as a function of increasing urban population; and vulnerability — due to increasing share of the elderly population.

We have also shown that well-known urban climate mitigation approaches, especially shading and street trees, could help in reducing the heat stress (although not eliminating it), and this is more advantageous in certain instances (such as N-S-oriented streets and in the afternoons). At the same time, the ‘best’ scenarios are currently not possible under the existing building regulations. Such regulations need to change to accommodate the thermal comfort realities being imposed on cities in Sri Lanka due to both urban and global warming.

Other actions (including behavioural adaptations such as avoiding outdoor working, especially at noontime, and heat advisories in the hottest months) need to accompany infrastructure changes. Given the rapidity of the trends, the window of opportunity to affect these changes is closing rapidly. Ultimately, the ‘best’ mitigation is to arrest the continuing global warming, which limits the ability of urban design and planning actions to minimise heat stress. Street design action could only modestly reduce the heat stress and that too, only for a limited time. Wider actions to tackle climate change itself are urgently needed.
